# Occurrence and antimicrobial resistance profile of *Listeria monocytogenes* isolated from chilled processed meat products in the Metropolitan Region of Recife, Brazil

**DOI:** 10.1007/s42770-026-02070-z

**Published:** 2026-09-04

**Authors:** Anízia Maria Vieira de Souza Lapenda, Eduarda Faria Raymundo, Pedro Marinho de Carvalho Neto, Rinaldo Aparecido Mota, José Wilton Pinheiro Junior, Andrea Paiva Botelho Lapenda de Moura

**Affiliations:** Universidade Federal Rural de Pernambuco, Recife, PE Brazil

**Keywords:** Listeriosis, Foodborne diseases, Processed meat, Sausage, Ham

## Abstract

The presence of *Listeria monocytogenes* in food represents a major challenge to food safety, especially in ready-to-eat products stored under refrigeration. This risk is associated with the ability of the pathogen to survive and multiply during prolonged refrigerated storage, making these foods potential sources of infection and a relevant public health concern. In this context, the aim of this study was to investigate the presence of *Listeria monocytogenes* in chilled processed meat products sold in bulk in the Metropolitan Region of Recife, PE, Brazil, as well as to evaluate the antimicrobial susceptibility profile of the isolates. A total of 48 samples of sausages and ham from different brands were analyzed, obtained from public markets and supermarkets in the region. Detection of *Listeria* spp. was performed using conventional methods recommended by the Ministry of Agriculture and Livestock, and antimicrobial susceptibility was assessed by the disk diffusion method. Of the analyzed products, 27/48 (56.25%) samples were positive for *Listeria* spp. by bacterial isolation. Among these, 15/48 (31.25%) were confirmed as *L. monocytogenes*. In the disk diffusion assay, one *L. monocytogenes* isolate showed a multidrug-resistant profile to vancomycin, amoxicillin, ampicillin, and ciprofloxacin. Additionally, among the *Listeria* spp. isolates, 6/27 (22.22%) showed resistance to at least one of the tested antimicrobials, with emphasis on ciprofloxacin (11.1%). These results demonstrate the presence of *Listeria* spp. and *L. monocytogenes* in commercially available chilled processed meat products, highlighting the importance of microbiological monitoring of these foods and surveillance of antimicrobial resistance profiles.

## Introduction

*Listeria monocytogenes* is a Gram-positive bacterium widely recognized as a foodborne pathogen responsible for listeriosis, an infection of high clinical severity. Infection occurs primarily through the ingestion of food contaminated with this microorganism, which is capable of affecting numerous mammals, including humans [1]. Although relatively rare, human listeriosis is associated with high hospitalization and mortality rates worldwide, particularly among high-risk groups such as pregnant women, newborns, the elderly, and immunocompromised individuals [2].

Species of the genus *Listeria* exhibit a high capacity to adapt to adverse environmental conditions, including low temperatures, oxygen availability, acidity, and high salt concentrations [3]. These characteristics favor their survival in refrigerated and ready-to-eat products, even in vacuum-packaged foods such as processed meat products, making them important vehicles of transmission. Contamination of these foods may occur from raw materials, through survival during technological processing, or via cross-contamination during steps such as slicing and handling. Ready-to-eat foods are the primary vehicles associated with the transmission of this pathogen to humans and have been implicated in major listeriosis outbreaks [4].

Infection by *L. monocytogenes* occurs through the consumption of contaminated food products, and its clinical presentation in humans may range from subclinical febrile gastroenteritis to severe invasive disease [4]. Outbreaks of listeriosis associated with contaminated food consumption have been reported in several countries, reinforcing the relevance of this pathogen to food safety and public health. In Brazil, although case reporting remains limited, studies have demonstrated the occurrence of *Listeria* spp. and *L. monocytogenes* in different food categories, indicating a potential risk to the population [1, 5].

In this context, the detection of *L. monocytogenes* is essential for the microbiological monitoring of foods, contributing to outbreak prevention, ensuring food safety, and protecting public health. Furthermore, the assessment of antimicrobial resistance profiles of *Listeria* spp. isolates has gained increasing attention, as although these bacteria are traditionally susceptible to antimicrobials used in the treatment of listeriosis, resistant strains have been reported in foods, environments, and clinical cases. This scenario underscores the need for continuous surveillance, given its potential impact on therapy and public health [6, 7].

Therefore, this study aimed to detect *Listeria* spp. and *L. monocytogenes* and to evaluate the antimicrobial resistance profile of isolates, obtained from chilled processed meat products commercialized in the metropolitan region of Recife, Pernambuco, Brazil.

## Materials and methods

### Sampling

A total of 48 chilled processed meat product samples were collected using convenience sampling from four different brands, comprising 24 hot dog–type sausage samples (brands A and B) and 24 light ham samples (brands C and D). The samples were purchased in bulk from public markets and supermarkets located in the Metropolitan Region of Recife, Pernambuco, Brazil. All samples were produced in establishments under Federal Inspection Service (SIF) and were within the manufacturer’s stated shelf life at the time of acquisition.

### Sample collection

Each sample, weighing approximately 300 g, was individually packaged in plastic bags at the point of purchase, placed in insulated containers containing reusable ice packs, and transported under refrigeration until the beginning of the analyses in the laboratory.

### Isolation of *Listeria* spp

Samples were analyzed using the conventional method based on ISO 11290-1 [8]. Initially, under a biological safety cabinet, the samples were identified through coding and subjected to aseptic weighing, with 25 g of each sample (ham and sausage) collected and placed into sterile bags. For the isolation of *Listeria* spp. using the conventional method, a selective enrichment step was first performed: each 25 g sample was homogenized with 225 mL of UVM broth (Difco) for 2 min and incubated in a bacteriological incubator at 30 °C for 24 h. Subsequently, 0.1 mL aliquots were transferred to tubes containing 10 mL of Fraser broth (Difco), supplemented with 0.1 mL of a 5% ammonium ferric citrate solution, and then incubated under the same conditions. After incubation, aliquots from the Fraser broth were streaked onto plates containing Tryptose Nalidixic Acid agar (TNA) and PALCAM agar (Difco), supplemented with polymyxin B sulfate. The plates were incubated at 30 °C for 24 h and 48 h, respectively.

### Biochemical identification of *Listeria* species

Suspected *Listeria* spp. colonies were subcultured into tubes containing nutrient agar and incubated at 36 °C for 24 h. After the incubation period, the cultures were subjected to Gram staining, catalase testing, and motility assessment at 25 °C. For identification tests, the reference strains *Listeria monocytogenes* (ATCC 19114), *Staphylococcus aureus* (ATCC 25923), and *Rhodococcus equi* were used. Carbohydrate fermentation tests (mannitol, rhamnose, and xylose) were performed, as well as evaluation of β-hemolysis on 8% sheep blood agar and CAMP test on Columbia agar (BBL) with *Staphylococcus aureus* (CAMP-SA) and *Rhodococcus equi* (CAMP-RE) [6].

### Antimicrobial susceptibility testing

All isolates were subjected to antimicrobial susceptibility testing using the disk diffusion method in accordance with Clinical and Laboratory Standards Institute guidelines, following CLSI M45 (3rd edition, 2015), with CLSI M100 (27th edition, 2017) used as supplementary reference [9, 10]. Inocula were prepared in Mueller–Hinton broth and incubated at 37 °C for 2 to 6 h, followed by adjustment of turbidity to the 0.5 McFarland standard. Subsequently, using sterile swabs, the suspensions were evenly spread over the entire surface of Mueller–Hinton agar plates and allowed to dry at room temperature for approximately 5 min.

Antimicrobial disks from the following classes were then applied: glycopeptides [vancomycin (30 µg)], aminoglycosides [gentamicin (10 µg) and streptomycin (300 µg)], β-lactams [ampicillin (10 µg) and amoxicillin (10 µg)], macrolides [erythromycin (15 µg)], tetracyclines [tetracycline (30 µg)], quinolones [ciprofloxacin], sulfonamides [cotrimoxazole] and amphenicols [chloramphenicol (30 µg)]. Plates were incubated at 37 °C for 16 to 18 h. After incubation, inhibition zones were measured using a millimeter ruler, and results were interpreted as susceptible, intermediate, or resistant.

### Statistical analysis

For statistical analysis, Fisher’s exact test was used, adopting a significance level of 5%. Analyses were performed using Epi Info software, version 7.2.3.1.

## Results

Of the 48 samples analyzed, 27 (56.25%) were positive for *Listeria* spp. isolation. Upon microbiological confirmation, 15/48 samples (31.25%) were identified as *L. monocytogenes*, 10/48 (20.83%) as *L. innocua*, and 2/48 (4.16%) as *L. welshimeri*. The occurrence of *Listeria* spp. was 16/27 (59.26%) in sausages and 11/27 (40.74%) in ham. Regarding the type of establishment, the frequency was 13/27 (48.15%) in both public markets and 14/27 (51.85%) supermarkets (Table 1).

No statistically significant difference was observed between sausages and ham regarding the occurrence of *Listeria* spp. (*p* > 0.05), nor between the types of establishments (*p* > 0.05). Similarly, no significant differences were found for *Listeria monocytogenes* between the products or among the evaluated establishments (*p* > 0.05).

**Table 1:** Frequency of *Listeria* spp. in chilled processed meat products, according to product type (sausage and ham) and type of establishment (public market and supermarket)

Variable	Category	*n*/*N* (%) *Listeria* spp.
Product	Sausage	16/27 (59.26%)
	Ham	11/27 (40.74%)
Establishment	Public market	13/27 (48.15%)
	Supermarket	14/27 (51.85%)

Among the *Listeria* spp. isolates, 6/27 (22.22%) showed resistance to at least one of the tested antimicrobials, with the highest resistance observed for ciprofloxacin (11.1%), followed by vancomycin (7.4%), amoxicillin (7.4%), ampicillin (7.4%), gentamicin (3.7%), and chloramphenicol (3.7%). Ciprofloxacin exhibited the highest frequency of resistance, corresponding to the lowest percentage of susceptibility (77.8%). In contrast, no resistance was observed to tetracycline and erythromycin, which showed 100% susceptibility (Table 2).

**Table 2:** Antimicrobial susceptibility profile of *Listeria* spp. isolates (*n* = 27) obtained from chilled processed meat products

Class	Antibiotics	Resistant *n*/*N* (%)	Intermediate *n*/*N* (%)	Susceptible *n*/*N* (%)
Aminoglycosides	Streptomycin	0/27 (0.0%)	1/27 (3.7%)	26/27 (96.3%)
Quinolones/Sulfonamides	Cotrimoxazole	0/27 (0.0%)	1/27 (3.7%)	26/27 (96.3%)
Glycopeptides	Vancomycin	2/27 (7.4%)	0/27 (0.0%)	25/27 (92.6%)
Tetracyclines	Tetracycline	0/27 (0.0%)	0/27 (0.0%)	27/27 (100.0%)
Aminoglycosides	Gentamicin	1/27 (3.7%)	1/27 (3.7%)	25/27 (92.6%)
β-lactams	Amoxicillin	2/27 (7.4%)	0/27 (0.0%)	25/27 (92.6%)
Amphenicols	Chloramphenicol	1/27 (3.7%)	1/27 (3.7%)	25/27 (92.6%)
β-lactams	Ampicillin	2/27 (7.4%)	0/27 (0.0%)	25/27 (92.6%)
Quinolones/Sulfonamides	Ciprofloxacin	3/27 (11.1%)	3/27 (11.1%)	21/27 (77.8%)
Macrolides	Erythromycin	0/27 (0.0%)	0/27 (0.0%)	27/27 (100.0%)

Regarding species distribution, among the 15 *Listeria monocytogenes* isolates, 5 showed resistance to at least one antibiotic (33.33%), whereas among the 10 *L. innocua* isolates, 1 exhibited antimicrobial resistance (10.0%). Notably, one *L. monocytogenes* isolate was identified with a multidrug-resistant profile, showing simultaneous resistance to vancomycin, amoxicillin, ampicillin, and ciprofloxacin (Table 3).

**Table 3:** Antimicrobial resistance profile of *L. monocytogenes* (*n* = 15) and *Listeria innocua* (*n* = 10) isolates obtained from processed meat products

Antibiotics	*L. monocytogenes*	*L. innocua*
Streptomycin	-	-
Cotrimoxazole	-	-
Vancomycin	LM1; LM2	-
Tetracycline	-	-
Gentamicin	LM3	-
Amoxicillin	LM2	LI1
Chloramphenicol	LM4	-
Ampicillin	LM2	LI1
Ciprofloxacin	LM5; LM2	LI1
Erythromycin	-	-
Total samples	5/15 (33.33%)	1/10 (10.0%)

*LM* *Listeria monocytogenes*; *LI Listeria innocua*

## Discussion

The high occurrence of *Listeria* spp. (56.25%) and *Listeria monocytogenes* (31.25%) observed in this study demonstrates the widespread dissemination of this pathogen in refrigerated processed meat products. These findings are particularly relevant because ham is typically consumed without further heat treatment, increasing the risk of exposure. Although hot dog-style sausages are usually cooked before consumption, cross-contamination may still occur during handling, storage, or preparation, particularly given the ability of *L. monocytogenes* to survive and proliferate under refrigeration. Furthermore, *L. monocytogenes* is considered the primary agent associated with human listeriosis, with meat products representing important transmission routes for this microorganism [4].

The results obtained are consistent with previous studies conducted in Brazil and other countries, which have reported the presence of *L. monocytogenes* in processed meat products [5–7, 10]. In the present study, a higher frequency of *Listeria* spp. was observed in sausages (16/27; 59.26%) compared to ham (11/27; 40.74%). However, variations in prevalence have been reported in the literature. For instance, Maia et al. [5] reported a lower prevalence in ham (13.75%; 11/80), while Ristori et al. [11] detected *L. monocytogenes* in 37.7% of sausage samples. These differences may be related to variations in hygienic–sanitary conditions, post-processing contamination, storage and commercialization practices, as well as differences in sampling design and methodologies employed.

The detection of non-pathogenic *Listeria* spp., such as *L. innocua* (20.83%) and *L. welshimeri* (4.16%), also deserves attention, as their presence is considered indicative of failures in hygienic–sanitary conditions and may reflect contamination of the processing environment [3]. The occurrence of these microorganisms in food is mainly associated with post-processing contamination, since *Listeria* spp. have a high capacity to persist in industrial environments and to form biofilms, favoring their survival even under adverse conditions such as refrigeration. Furthermore, these species share ecological niches with *L. monocytogenes* and may act as indicators of an environment conducive to the presence of this important pathogen. In this context, the high frequencies observed may indicate the occurrence of cross-contamination throughout the production chain [12].

Although no statistically significant differences were observed among the types of food, establishments, or brands analyzed (*p* > 0.05), the detection of the pathogen across all groups suggests contamination distributed throughout the production and commercialization chain of bulk products. This pattern reinforces the hypothesis of multiple critical points of contamination, including processing, slicing, and handling stages, particularly in foods handled at the point of sale. Studies have indicated a higher frequency of *L. monocytogenes* in products handled at retail compared to those pre-packaged by the manufacturer and not subjected to additional handling [13, 14].

Regarding the antimicrobial susceptibility profile, a high frequency of antimicrobial-resistant isolates (22.22%) was observed, particularly among *Listeria monocytogenes*, corroborating previous studies reporting that this species has developed resistance to first-line antimicrobials used in the treatment of listeriosis, such as ampicillin and amoxicillin [6, 7, 15]. This scenario is of public health concern, given that aminopenicillins, commonly combined with gentamicin, constitute the standard treatment for invasive listeriosis in humans. Furthermore, therapeutic options are further constrained by the intrinsic resistance of *L. monocytogenes* to cephalosporins. Nevertheless, all isolates were 100% susceptible to erythromycin and tetracycline, therapeutic alternatives considered in specific contexts, such as β-lactam allergy [16].

In the present study, the highest frequency of antimicrobial resistance was observed for ciprofloxacin, which, although not part of the first-line therapeutic regimen for listeriosis, may indirectly reflect the excessive use of this antibiotic in the treatment of multiple infections. In *L. monocytogenes*, fluoroquinolone resistance is mediated by efflux pumps, most notably the Lde pump, belonging to the Major Facilitator Superfamily (MFS), and FepA, a member of the Multidrug And Toxic compound Extrusion (MATE) family. Point mutations in the regulatory gene *fepR* result in FepA overexpression, conferring high-level resistance to ciprofloxacin [16, 17]. The presence of this mechanism in the isolates of the present study, although not confirmed by molecular analysis, cannot be ruled out.

Concurrently, a multidrug-resistant (MDR) *L. monocytogenes* isolate (LM2) resistant to vancomycin, amoxicillin, ampicillin, and ciprofloxacin was identified. Notably, vancomycin resistance has rarely been documented in food or clinical isolates of *L. monocytogenes*, making this a striking finding within the identified MDR profile. This resistance profile is alarming, as it simultaneously compromises first-line therapeutic options and potential alternatives. The detection of MDR isolates in food is an epidemiological concern, since resistance in *L. monocytogenes* is driven by horizontal gene transfer mediated by mobile genetic elements such as plasmids and transposons, which facilitate dissemination of resistance genes across strains and species, alongside efflux-mediated mechanisms as previously described [16]. The recent increase in reports of MDR *L. monocytogenes* in the literature [6, 7] suggests an emerging resistance trend, reinforcing the public health relevance of the present findings.

These findings may be associated with selective pressure resulting from the intensive use of antimicrobials in human health and animal production, as well as with conditions in food processing environments that favor the dissemination of resistant strains In this context, these data highlight the need for continuous monitoring of *Listeria* spp. in ready-to-eat foods, combined with the rigorous implementation of good manufacturing practices and effective sanitary control measures [18].

A limitation of this study is that species identification was based solely on phenotypic and biochemical methods, without molecular confirmation by PCR, which may affect species-level resolution among closely related *Listeria* spp., although these approaches are standard in routine food microbiology for preliminary identification. Additionally, the relatively small sample size may limit the generalizability of the findings. Future studies should therefore combine larger sample collections with molecular methods to improve both representativeness and species-level accuracy.

## Conclusion

This study demonstrated the occurrence of *Listeria* spp., including *Listeria monocytogenes*, in processed meat products intended for human consumption. Additionally, isolates exhibiting antimicrobial resistance, including multidrug resistance, were detected, indicating a potential risk to public health and highlighting the importance of microbiological monitoring of these foods and surveillance of antimicrobial resistance profiles.
